# Nuclear translocation of thioredoxin-1 promotes colorectal cancer development via modulation of the IL-6/STAT3 signaling axis through interaction with STAT3

**DOI:** 10.7150/thno.85460

**Published:** 2023-08-28

**Authors:** Aihua Wu, Daoquan Fang, Yangyang Liu, Xiaomeng Shi, Zuyue Zhong, Baojian Zhou, Lechi Ye, Xuecheng Sun, Lei Jiang

**Affiliations:** 1 Department of Laboratory Medicine, The First Affiliated Hospital of Ningbo University, Ningbo 315010, China.; 2 Central Laboratory, The First Affiliated Hospital of Wenzhou Medical University, Wenzhou 325000, China.; 3 Department of Colorectal and Anal Surgery, The First Affiliated Hospital of Wenzhou Medical University, Wenzhou 325000, China.; 4 Department of Gastroenterology, the First Affiliated Hospital of Wenzhou Medical University, Wenzhou 325000, China.

**Keywords:** Colorectal cancer, IL-6, nuclear translocation, STAT3, Trx-1

## Abstract

**Background:** Thioredoxin 1 (Trx-1) is a small redox protein predominantly localized in the cytoplasm. Its expression is increased in several cancers, including colorectal cancer (CRC). However, the function of Trx-1 translocation to the nucleus in cancer is not clear. In this study, we investigated the role of Trx-1 nuclear translocation in development of CRC.

**Methods:** Expression of Trx-1 and STAT3 was analyzed by Western blot and immunofluorescence. Endogenous interaction of Trx-1, STAT3, and karyopherin α1 in CRC cells was analyzed by co-immunoprecipitation. Trx-1 and pSTAT3 nuclear staining in human CRC tissues was analyzed by immunohistochemistry. A mouse model of AOM/DSS induced colitis-associated cancer (CAC) was utilized to investigate the antitumor effect of PX-12, a Trx-1 inhibitor. A knockin mouse with the *Txn1*(KK81-82EE) mutation was generated via CRISPR/Cas9, and CAC was induced in knockin and wild-type mice.

**Results:** Nuclear translocation of Trx-1 was induced by IL-6, and inhibition of this translocation reversed IL-6-induced epithelial-to-mesenchymal transition, invasion and metastasis. Karyopherin α1 was found to specifically mediate IL-6-induced translocation of the Trx-1-pSTAT3 complex into the nucleus. Nuclear Trx-1 expression was closely correlated with lymph node metastasis and distant metastasis in human CRC. In addition, nuclear staining of Trx-1 showed significant positive correlation with nuclear staining of pSTAT3 in human CRC tissues. PX-12, an inhibitor of Trx-1, significantly impaired the activation of STAT3 and suppressed the development of AOM/DSS-induced CAC in mice. Moreover, AOM/DSS-induced nuclear Trx-1 expression was suppressed in *Txn1*(KK81-82EE) mice, which inhibited STAT3 activation and cancer progression.

**Conclusions:** These results provide new insights into the mechanisms of STAT3 activation triggered by IL-6 and identify nuclear translocation of Trx-1 as a potential therapeutic target for the treatment of CRC and CAC.

## Introduction

It is becoming increasingly clear that chronic inflammation increases the risk of cancer as well as facilitates tumor progression and metastasis 1, 2. Chronic inflammatory bowel disease is a firmly established risk factor for the type of colorectal cancer (CRC) known as colitis-associated cancer (CAC) 3. In particular, patients with long-standing ulcerative colitis (UC) have a higher risk of developing CRC, with an incidence of up to 20% within 30 years of UC onset 4. Inflammatory bowel disease is characterized by abnormal production of proinflammatory cytokines and heavy infiltration of immune cells, which promote the development and progression of malignancies 5, 6.

Interleukin 6 (IL-6) is an important cytokine secreted by cells that infiltrate the tumor microenvironment, such as tumor-associated macrophages, which are thought to promote tumor growth 7, 8. Broadly, IL-6 and its intracellular signaling molecule signal transducer and activator of transcription 3 (STAT3) promote the proliferation, motility, and invasiveness of cancer cells 9, 10. IL-6/STAT3 is known to regulate epithelial-to-mesenchymal transition (EMT), a crucial process in tumor metastasis and invasiveness 11, 12. Aberrant expression of IL-6/STAT3 has also been shown to contribute to tumor aggressiveness and lower survival in CRC patients and AOM/DSS-induced CAC model mice 13-15. Induction of STAT3 by cytokines or growth factors leads to its constitutive activation and phosphorylation at tyrosine residue 705. pSTAT3^Tyr705^ forms a dimer and is imported into the nucleus, where it then binds to target genes and regulates transcriptional activity 12, 16. IL-6 is also able to induce nuclear transport of some specialized proteins, which could lead to genetic changes in cells 17.

Thioredoxin-1 (Trx-1) is a small and ubiquitous protein with multiple functions including antioxidant and redox regulation, modulation of transcription factor activity, and regulation of apoptosis 18-20. Trx-1 protein is predominantly located in the cytoplasm in humans, where it interacts with multiple proteins, including inhibiting apoptosis induction 21. Trx-1 has been reported to increase IL-6 synthesis in a dose-dependent manner in fibrosarcoma and endothelial cells 22. In addition, the Trx system regulates the STAT3 pathway via directly regulating the redox state of STAT3 and indirectly regulating the redox states of peroxiredoxin or SHP 19, 23. Many Trx/TrxR inhibitors have been found to promote STAT3 oxidation and block STAT3 activation 24, 25. Accordingly, it suggested that the Trx system may be important in regulating the activity of STAT3-related signaling pathways, thus affecting STAT3-dependent transcription 19. We have previously reported that in human CRC, increased Trx-1 expression significantly correlates with clinical stage, lymph node metastasis, and poor survival 26. We also found that Trx-1 promotes EMT-mediated CRC invasion and metastasis by upregulating S100A4 via crosstalk with S100P 26, 27. Specifically, Trx-1 promotes transcription of *S100P* gene by increasing the DNA binding activity of SP1, and S100P in turn induces Trx-1 expression and nuclear translocation through upregulation of p-ERK1/2 and downregulation of TXNIP expression 26.

Under certain stress conditions, including hydrogen peroxide (H_2_O_2_) treatment, nitric oxide exposure, ultraviolet irradiation, and viral infection, Trx-1 is translocated to the nucleus where it binds to several transcription factors, such as NF-kappa B and AP-1, to enhance their transcriptional activity and expression 28-30. Thus, nuclear Trx-1 has an important function as resistance to apoptosis and oxidation. This study further determined that IL-6-induced Trx-1 nuclear translocation plays critical roles in EMT, invasion and metastasis of CRC cells, and progression of CAC through an interaction of Trx-1 with STAT3, with karyopherin α1 specifically strengthening the Trx-1-STAT3 complex to accelerate its nuclear translocation.

## Materials and Methods

### Cell lines and reagents

Human CRC cell lines HT-29 and SW480 were obtained from the Cell Bank of Chinese Academy of Sciences (Shanghai, China). HT-29 cells were cultured in McCoy's 5a Modified Medium supplemented with 10% fetal bovine serum (Invitrogen, Carlsbad, CA, USA). SW480 cells were cultured in L-15 medium (Sigma-Aldrich, St. Louis, MO, USA) supplemented with 10% fetal bovine serum. The STAT3 inhibitor S3I-201 and Trx-1 inhibitor PX-12 (1-methylpropl 2-imidazolyl disulfide) were purchased from Selleck Chemical (Shanghai, China). Recombinant human IL-6 was purchased from R&D Systems (Minneapolis, MN, USA) and reconstituted at a concentration of 100 μg/mL in sterile phosphate buffer saline containing 0.1% bovine serum albumin.

### Plasmid, lentivirus production and transduction

Lentiviral vectors expressing enhanced green fluorescent protein (GFP, as control) and human wild-type (WT) *Trx-1* gene, as well as shRNA targeting human *Trx-1* gene (shTrx-1, targeting sequence: 5'- GACTGTCAGGATGTTGCTTCAGAGTGTGA -3') were generated in our previous work 26. A mutant (MT) *Trx-1* (KK81-82EE, MT-Trx-1) was generated by site-directed mutagenesis using PCR-driven overlap extension 31. Briefly, the reaction requires flanking primers complementary to the ends of the *Trx-1* sequence ( forward primer A with a EcoR Ⅰ site: 5'- CGAATTCGCCACCATGGTGAAGCAGATCGAGAGCAA -3', and reverse primer D with a Sac Ⅱ site: 5'- GCGCCGCGGTTAGACTAATTCATTAATGGTGGCT -3'), and two internal primers with complementary ends that containing the desired mutation and generating overlapping nucleotide sequences (reverse primer B: 5'- CCAACATTCCAGTTTTTTGAGGAGGGACAAAAGGTGGGTG -3' and forward primer C: 5'- CACCCACCTTTTGTCCCTCAAAAAACTGGAATGTTG -3'). The AB and CD PCR fragments were amplified in the first round of PCR, then used as template for primers A and D in the second round of PCR. The final product AD was inserted into a lentiviral vector to generate the lenti-MT-Trx-1 vector containing mutant *Trx-1* (KK81-82EE, MT-Trx-1). Lentivirus production and transduction was performed according to previous protocols 26.

### Western blot analysis

Total cell lysate, cytoplasmic protein lysate, and nuclear protein lysate were prepared in RIPA lysis buffer or in cytoplasmic and nuclear protein lysis buffer with protease and phosphatase inhibitor (Thermo Scientific, Rockford, USA). Western blot analysis was performed as previously described 27. Primary antibodies reactive to E-cadherin (1:4 000, BD; Cat#610181), vimentin (1:2 000, BD, Cat#550513), β-actin (1:1 000, Cell Signaling Technology, Danvers, MA, USA; Cat#4970), Trx-1 (1:10 000, Abcam, Cambridge, UK, Cat#ab133524), STAT3 (1:2 000, Cell Signaling Technology, Cat#9139), Phospho-STAT3 Tyr705 (1:1 000, Cell Signaling Technology, Cat#9145), Lamin B1 (1:1 000, Bioworld, St. Louis Park, MN, USA, Cat#BS3547), karyopherin α1(1:500, Santa Cruz Biotechnology, Santa Cruz, CA, USA, Cat#sc-101292), and IL-6 (1:500, Santa Cruz, Cat#sc-1265) were used.

### Co-immunoprecipitation (IP)

A co-IP assay was used to determine the interaction between Trx-1, STAT3, and karyopherin α1. Briefly, 500 μg of total protein, 250 μg of nuclear protein, or 500 μg of cytoplasmic protein was incubated with 30 μL of protein G beads (Bio-Rad Laboratories, Hercules, California, USA, Cat #161-4823) and 1 μg of the indicated antibody in a rotary vibrator for 1 h at room temperature. Afterwards, the beads were eluted with 40 μL of 1 × Laemmli sample loading buffer, and proteins analyzed by Western blotting. The primary antibodies used were anti-Trx-1 (1:100, Abcam), anti-pSTAT3^Y705^ (1:100, Cell Signalling Technology), anti-STAT3 (1:200, Cell Signalling Technology), and anti-karyopherin α1 (1:30, Santa Cruz Biotechnology).

### SiRNAs transfection

The siRNA target sequence for human STAT3 is 5'- CAGCCTCTCTGCAGAATTCAA -3' and siRNA target sequence for karyopherin is 5'- TGGAGTTCCTCAAACGAAATT -3'. SiRNAs were transfected into CRC cells using Lipofectamine 3000 (Invitrogen) according to the manufacturer's instructions.

### Migration and invasion assay

We conducted migration and invasion assays using Transwell cell culture chamber inserts with 8 μm pores (Corning Costar Corp, Cambridge, MA, USA). For the migration assay, 5 × 10^5^ cells were suspended in 200 μL serum-free medium and plated in the upper chamber. For the invasion assay, the chamber inserts were coated with 10 μL Matrigel (BD), and then 5 × 10^5^/200 μL cells were plated in the upper chamber. Medium containing 20% FBS as chemoattractant was added into the lower chamber. After an incubation period of 48 h, the cells on the membrane were fixed with formalin and stained with 0.5% crystal violet. The nonmotile cells in the upper surface of the membrane were carefully removed and the migrating and invading cells were visualized and counted under a microscope (Olympus).

### Immunofluorescence

Cells were grown on a coverslip and fixed at 4 °C for 20 min with 4% paraformaldehyde. After permeabilization with 0.3% Triton X-100, the cells were blocked overnight at room temperature with 5% BSA. Subsequently, the cells were incubated with the indicated primary antibodies (anti-Trx-1, 1:200, Abcam; and anti-STAT3, 1:100, Cell Signaling Technology) at 4 °C overnight. After washing, the cells were stained using a second antibody (Alexa-Fluor® 488 goat anti-mouse antibody IgG or Alexa- Fluor® 594 goat anti-rabbit antibody IgG, 1:1 000, Invitrogen) and 4'-6-diamidino-2-phenylindole (DAPI, Life Technologies).

### RNA isolation and real-time PCR

Total RNA was extracted using TRIzol reagent (Invitrogen), and cDNA was generated using the RevertAid First Strand cDNA Synthesis Kit (Thermo Scientific) according to the manufacturer's instructions. Real-time PCR was performed using SYBR Green Master Mix (Takara, Japan) and the 7500-Real-Time PCR System (Applied Biosystems). The relative mRNA levels for each gene were determined by the comparative CT method (ΔΔCT method) with GAPDH as an internal control. The PCR primer sequences are given in **Supplementary Table S1**.

### Immunohistochemistry (IHC)

For IHC, tissue paraffin sections were deparaffinized in xylene and rehydrated in descending concentrations of ethanol and then interdict with H_2_O_2_. Antigen retrieval was performed by microwave heating in citrate or EDTA buffer. Sections were blocked with 5% goat serum before incubation overnight with primary antibodies. Subsequently, the sections were incubated with biotinylated secondary antibodies, then stained with DAB and hematoxylin for counterstaining, dehydrated in ascending concentrations of ethanol and xylene, and finally preserved in mounting medium. Primary antibodies were anti-Trx-1 (1:500, Abcam, Cat#ab26320), anti-STAT3 (1:100, Cell Signaling Technology, Cat#9139), anti-phospho-STAT3 Y705 (1:500, Abcam, Cat#ab76315), anti-E-cadherin (1:200, Cell Signaling Technology, Cat#3195), anti-vimentin (1:500, BD, Cat#550513), anti-Ki-67 (1:100, Abcam, Cat#ab16667). For H&E staining, 5 μm tissue sections were stained with eosin and hematoxylin.

### Human CRC samples

Fresh human CRC samples (n = 48) were collected from patients who had undergone surgical resection at the First Affiliated Hospital of Wenzhou Medical University after giving informed consent. In addition, a total of 157 CRC cases were retrospectively selected from the surgical pathology database of the First Affiliated Hospital of Wenzhou Medical University, China. All cases underwent colectomy and had not received chemotherapy or radiotherapy before surgery. Their tissue paraffin sections were analyzed by IHC. This study was approved by the Ethics Committee of the First Affiliated Hospital of Wenzhou Medical University.

### CAC induction and PX-12 treatment in mice

Specific-pathogen-free six weeks old C57BL/6 male mice were provided by Shanghai Slaccas Animal Centre. Colitis-associated cancer model was induced by intraperitoneal injection of 12.5 mg/kg azoxymethane (AOM, Sigma-Aldrich) followed by three consecutive rounds of 2% dextran sodium sulfate (DSS, MP-Bio) provided in drinking water (7 days per round, 14 days interval between rounds). For PX-12 treatment, mice were injected with 12.5 mg/kg PX-12 (dissolved in 10% ethanol and 0.9% NaCl) into the tail vein after the first DSS administration and daily during the three recovery periods. Eleven weeks after AOM treatment, the mice were sacrificed and the entire colon of each mouse was removed. The coelenteron was carefully opened longitudinally, and the number of tumors in each colon was counted. A vernier caliper was used to measure each tumor nodule. Paraffin-embedded colon tissue sections were immunostained with the indicated antibodies. All the animal experiments were approved by the Laboratory Animal Ethics Committee of the First Affiliated Hospital of Wenzhou Medical University.

### Generation of *Txn1* (KK81-82EE)-knockin mice

*Txn1*(KK81-82EE)-knockin mice were generated using CRISPR/Cas9 technology and support from Cyagen Biosciences (Suzhou, China). Briefly, a sgRNA targeting *Txn1* exon 4 was designed with the following sequence: 5'- TTTTATAAAAAGGGTCAAAAGG -3'. A donor oligo containing the KK81-82EE (AAAAAG to GAGGAG) mutation was also designed with the sequence: 5'- GCATCCCAGGATGTTGCTGCAGACTGTGAAGTCAAATGCATGCCGACCTTCCAGTTTTATGAGGAGGGTCAAAAGGTACGTAGATCTCGATTTAAGAGCACCAGCTGGAGCTGGGCATGGTGATGC -3'. This oligo was used to introduce the mutation into exon 4 by homology-directed repair. Female C57BL/6 mice were superovulated, the oocytes harvested and fertilized, and the resulting zygotes co-injected with Cas9 mRNA, the sgRNA and the donor oligo to generate knockin mice. Pups were genotyped by PCR, followed by sequence analysis using the following primers: 5'- AACAGTGCAGTGAATAGCCACTGCAT -3' (sense) and 5'- CGTGCGTGCTCAACA CCCAG -3' (antisense), which produced a 525-bp product. Subsequent confirmation by sequence analysis used the sequencing primer: 5'- CGTGCGTGCTCAACACCCAG -3'. Positive founders were bred to produce the next generation (F1), which pups were again genotyped by PCR and DNA sequencing analysis to identify mutant *Txn1*(KK81-82EE) offspring. However, few homozygous knockin mice were generated, possibly because of mortality caused by homozygous mutations, so heterozygous mice were used in our study.

### Statistical analysis

All data from the experiments were analyzed using the GraphPad Prism 8.0 or SPSS software. Statistical data analysis was performed using the two-tailed Student's *t*-test, one-way ANOVA grouped analyses, or the Pearson correlation test, and *P* < 0.05 was considered statistically significant.

## Results

### IL-6 induces EMT, invasion, and metastasis of CRC cells by regulating nuclear translocation of Trx-1

To determine the effects of IL-6 on EMT, invasion and metastasis of CRC cells, HT-29 and SW480 cells were treated with recombinant IL-6. As expected, treatment with IL-6 resulted in a decrease in E-cadherin and an increase in vimentin, observed by immunofluorescence staining and Western blot analysis (**Figure S1A and B**). Transwell and wound healing assays showed that IL-6 induced the migration and invasion of CRC cells *in vitro* (**Figure S1C and D**); moreover, *ex vivo* treatment with IL-6 promoted formation of metastases in the lungs of NOD/SCID mice after injection of SW480 cells into the tail vein (**Figure S1E**). These results suggest that IL-6 promotes EMT, migration and invasion of CRC cells *in vitro* and metastasis *in vivo*.

**Figure 1:**
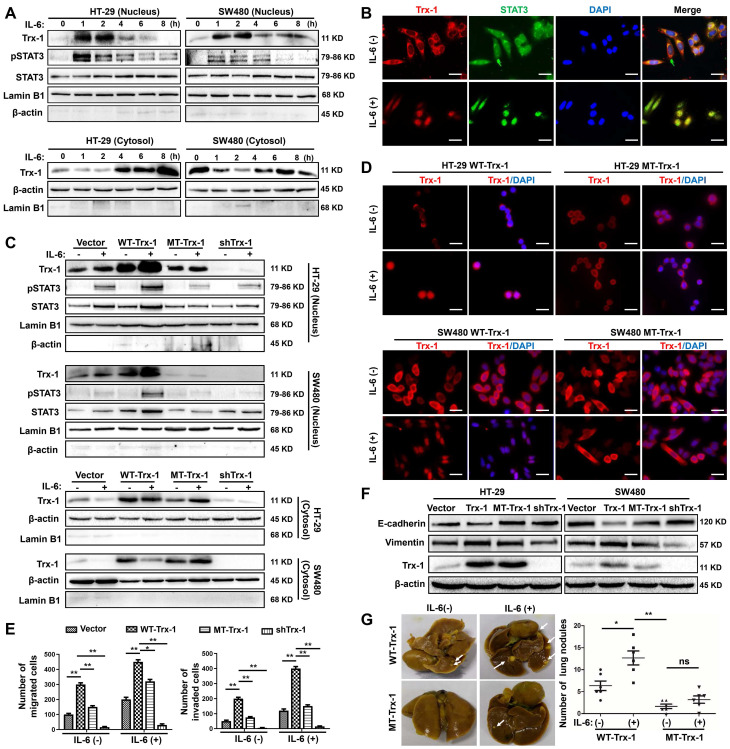
** Nuclear translocation of Trx-1 is required for IL-6-induced epithelial-to-mesenchymal transition (EMT), invasion and metastasis.** (**A**) IL-6 treatment induces translocation of Trx-1 from the cytosol to the nucleus, accompanied by increased pSTAT3 in the nucleus. Cytoplasmic and nuclear levels of Trx-1 and pSTAT3 were determined by Western blotting in SW480 and HT-29 cells treated with 20 ng/mL IL-6 for the indicated times. (**B**) Monitoring of Trx-1 and pSTAT3 localization by immunostaining in SW480 cells treated with 20 ng/mL IL-6 for 2 h. Cells were stained with anti-Trx-1 antibody (red) or anti-pSTAT3 (green), and with DAPI (blue) to localize the nucleus. Scale bar, 20 μm. (**C**) Protein levels as determined by Western blotting in nuclear and cytosolic extracts of HT-29 and SW480 cells stably expressing control vector, WT-Trx-1, MT-Trx-1, or shTrx-1 and treated with 20 ng/mL IL-6 for 2 h. (**D**) Monitoring of Trx-1 localization by immunostaining in HT-29 and SW480 cells stably expressing WT-Trx-1 or MT-Trx-1 and treated for 2 h with 20 ng/mL IL-6. Scale bar, 20 μm. (**E**) Migration and invasion capabilities of SW480 cells expressing control vector, WT-Trx-1, MT-Trx-1, or shTrx-1, detected by Transwell assay in the presence of IL-6 (20 ng/mL) for 48 h. (**F**) Total protein extracts from HT-29 and SW480 cells stably expressing control vector, WT-Trx-1, MT-Trx-1 or shTrx-1 were subjected to Western blotting. (**G**) Formation of lung metastases in NOD/SCID mice 8 weeks after tail-vein injection of SW480 cells expressing WT-Trx-1 or MT-Trx-1 with or without IL-6 pretreatment (5 days). Representative images of whole lungs are shown, along with quantifications of microscopic pulmonary nodules per mouse. Error bars represent S.E.M. *P < 0.05, **P < 0.01, ***P < 0.001. ns, not significant; WT-Trx-1, wild type *Trx-1* gene; MT-Trx-1, mutant type *Trx-1* gene (*Trx-1* KK81-82EE); shTrx-1, shRNA targeting *Trx-1*.

We further analyzed the localization of Trx-1 in SW480 and HT-29 cells by immunofluorescence after treatment with IL-6 at the indicated time points. In untreated SW480 and HT-29 cells, Trx-1 protein was mainly localized in the cytoplasm (**Figure S2A**). After cells were exposed to IL-6, most of the fluorescence shifted from the cytoplasm to the nucleus, with the maximal effect being observed after 2 h (**Figure S2A**). Western blot analysis was used to quantify cytoplasmic and nuclear Trx-1 protein levels. Consistent with the immunofluorescence staining results, the abundance of Trx-1 in the nuclear fraction extracts was greatly increased as early as 1 h after IL-6 stimulation, whereas the level in the cytoplasm decreased (**Figure 1A**). A maximal level of nuclear Trx-1 protein was observed after 2 h, with increases of approximately 4.5- and 2.5-fold in SW480 and HT-29 cells, respectively (**Figure S2B**). Subsequently, nuclear Trx-1 levels decreased, returning to the nonstimulated level after 8 h of exposure to IL-6 (**Figure S2B**). Analysis of the ratio of nuclear to cytosolic Trx-1 also showed that the peak level was reached 2 h after stimulation with IL-6, with increases 10- and 6.8-fold in SW480 and HT29 cells, respectively (**Table S2**).

We then examined whether IL-6-induced Trx-1 nuclear translocation is associated with activation of STAT3, an effector of IL-6 pathway. As expected, STAT3 was phosphorylated by IL-6 treatment, and IL-6 stimulated CRC cells exhibited maximal levels of nuclear p-STAT3 after 1 h of exposure, which decreased after 4 h (**Figure 1A**). As illustrated in **Figure 1B**, immunofluorescence staining confirmed that both Trx-1 (red) and STAT3 (green) were mainly localized in the cytoplasm before treatment, and translocate to the nucleus in response to IL-6 stimulation.

To investigate the possible role of Trx-1 nuclear translocation in IL-6-mediated EMT, invasion and metastasis of CRC, we used lentiviral transduction to establish SW480 and HT-29 cells stably expressing the control vector, WT-Trx-1 (wild-type *Trx-1* gene), MT-Trx-1 (KK81-82EE mutant *Trx-1*,), or shTrx-1 (shRNA targeting *Trx-1*). The *Trx-1* KK81-82EE mutant is reported to be nuclear-translocation-disabled due to the mutation altering the nuclear translocation sequence 32. As shown in** Figure 1C**, compared to the control, nuclear translocation of Trx-1 and pSTAT3 was enhanced by treatment with IL-6 in SW480 and HT-29 cells expressing WT-Trx-1 but prevented in cells expressing MT-Trx-1. Moreover, knockdown of Trx-1 resulted in inhibition of IL-6-induced nuclear translocation of pSTAT3. Immunofluorescence staining likewise showed IL-6-induced nuclear translocation of Trx-1 to be inhibited in SW480 and HT-29 cells expressing MT-Trx-1, but not in cells expressing WT-Trx-1 (**Figure 1D**). *In vitro* migration and invasion of these cells were also examined, revealing both activities to be increased by overexpression of Trx-1 in SW480 cells but comparatively decreased in cells expressing MT-Trx-1, with or without IL-6 stimulation (**Figure 1E and Figure S3**). When CRC cells were transduced with shTrx-1, the migration and invasion stimulated by IL-6 were substantially prevented (**Figure 1E and Figure S3**). Overexpression of WT-Trx-1 induced EMT (decrease in E-cadherin and increase in vimentin), whereas overexpression of MT-Trx-1 had no effect on EMT marker expression in CRC cells (**Figure 1F**), and silencing of Trx-1 inhibited EMT (increased E-cadherin and decreased vimentin, **Figure 1F**). Treatment with IL-6 facilitated metastasis formation in SW480 cells expressing WT-Trx-1, but this effect was substantially prevented in cells expressing MT-Trx-1 (**Figure 1G and Table S3**). All told, our results clearly demonstrate that IL-6 treatment induces EMT, invasion, and metastasis of CRC cells by regulating the nuclear translocation of Trx-1. Moreover, we performed STAT3 ChIP-seq and RNA-seq in control-vector, Trx-1, or shTrx-1-transfected SW480 cells. As shown in **Figure S4A**, the proximity of STAT3 peaks in the TSS region were detected and enriched in cells transfected with Trx-1 and abolished in cells transfected with shTrx-1. Overlap of changes in STAT3 occupancy with changes in mRNA levels measured by RNA-seq showed that changes in the STAT3 ChIP-seq coverage often lead to transcription alteration (**Figure S4B**).

### IL-6 induction of Trx-1 nuclear translocation depends on STAT3 activation

**Figure 2:**
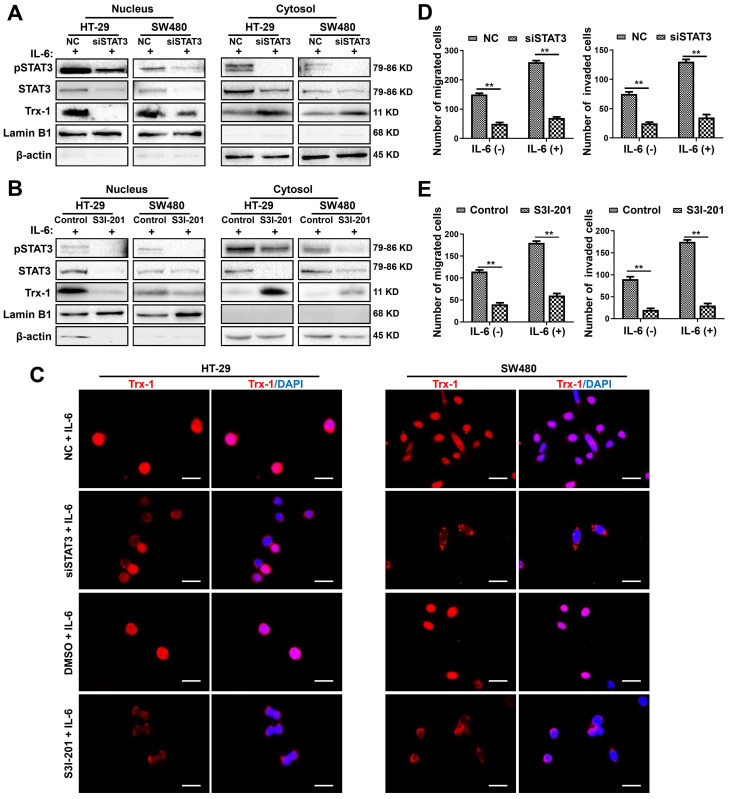
** Inhibition of the IL-6-STAT3 pathway blocks IL-6-induced Trx-1 nuclear translocation, migration and invasion.** (**A**) Cytoplasmic and nuclear levels of pSTAT3, STAT3 and Trx-1 determined by Western blotting in HT-29 and SW480 cells transfected with 50 μM STAT3-siRNA or NC siRNA for 48 h and then treated with IL-6 (20 ng/mL) for 2 h. (**B**) Cytoplasmic and nuclear levels of pSTAT3, STAT3 and Trx-1 determined by Western blotting in HT-29 and SW480 cells exposed to S3I-201 for 1 h and then treated with IL-6 for 2 h. (**C**) Trx-1 localization by immunostaining in HT-29 and SW480 cells transfected with 50 μM STAT3-siRNA or negative control (NC) for 48 h, or treated with S3I-201 for 1 h, followed by IL-6 treatment for 2 h. Cells were stained with anti-Trx-1 and DAPI. Scale bar, 20 μm. (**D**) Effect of STAT3 knockdown on the migration and invasion ability of CRC cells. SW480 cells were transfected with 50 μM STAT3-siRNA or NC for 48 h, and a Transwell assay performed in the presence of IL-6 (20 ng/mL) for 48 h. (**E**) Effect of Trx-1 inhibition on the migration and invasion ability of CRC cells. HT-29 and SW480 cells were exposed to S3I-201 for 1 h, and then a Transwell assay performed in the presence of IL-6 (20 ng/mL) for 48 h. The histogram gives the number of migrating and invading cells. **P < 0.01.

To further investigate the effect of IL-6/STAT3 on Trx-1 expression and nuclear translocation, we knocked down endogenous IL-6 or STAT3 expression using siRNAs. Knockdown of IL-6 in HT-29 and SW480 cells significantly inhibited STAT3 and Trx-1 protein expression (**Figure S5A**). Similar results were observed in HT-29 and SW480 cells transfected with STAT3 siRNA (siSTAT3) (**Figure S5B**). Moreover, knockdown of STAT3 reduced nuclear Trx-1 in the presence of IL-6, whereas cytoplasmic Trx-1 was increased (**Figure 2A**). We also treated HT-29 and SW480 cells with the STAT3 inhibitor S3I-201. As shown in **Figure 2B**, the specific inhibition of pSTAT3 resulted in loss of Trx-1 localization to the nucleus. Immunofluorescence analysis also showed the IL-6-induced redistribution of Trx-1 to be largely inhibited when HT-29 and SW480 cells were treated with siSTAT3 or S3I-201 (**Figure 2C**). Moreover, inhibition of STAT3 by siRNA or S3I-201 suppressed the migration and invasion ability and also reversed IL-6-induced migration and invasion in HT-29 and SW480 cells (**Figure 2D-E**). These observations suggest that STAT3 activation mediates IL-6-induced Trx-1 nuclear translocation, migration and invasion of CRC cells.

### Trx-1 interacts with STAT3 and its nuclear import is mediated by karyopherin α1

**Figure 3:**
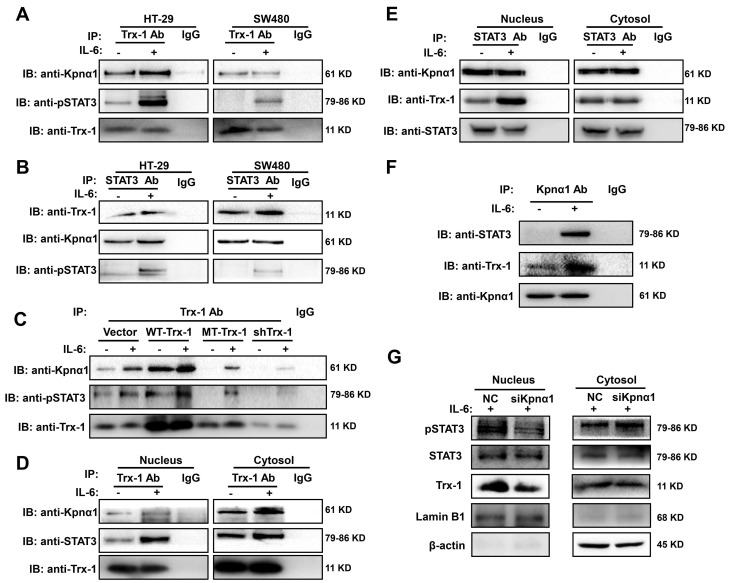
** STAT3 and Trx-1 interaction and nuclear import upon IL-6 stimulation is mediated by karyopherin α1 (Kpnα1).** (**A**) pSTAT3 and Kpnα1 co-immunoprecipitated with Trx-1. HT-29 and SW480 cells were treated with 20 ng/mL IL-6 or vehicle for 2 h and then subjected to co-immunoprecipitation (co-IP) with anti-Trx-1 antibody or control IgG, followed by immunoblotting with anti-pSTAT3 or anti-Kpnα1 antibody. (**B**) Trx-1 and Kpnα1 co-immunoprecipitated with STAT3. HT-29 and SW480 cells were treated with 20 ng/mL IL-6 or vehicle for 2 h and then subjected to co-IP with anti-STAT3 antibody or control IgG, followed by IB with anti-pSTAT3, anti-Kpnα1, or anti-Trx-1 antibody. (**C**) Effect of Trx-1 perturbation on protein interactions. Lysates of HT-29 cells expressing WT-Trx-1, MT-Trx-1, shTrx-1 or a control vector without or with IL-6 treatment were subjected to co-IP with anti-Trx-1 antibody, followed by IB with anti-pSTAT3 or anti-Kpnα1. (**D**) Trx-1 interactions according to cell compartment. Nuclear and cytosolic extracts of HT-29 cells without or with IL-6 treatment were subjected to co-IP with anti-Trx-1, followed by IB with anti-pSTAT3 or anti-Kpnα1. (**E**) STAT3 interactions according to cell compartment. Nuclear and cytosolic extracts of HT-29 cells without or with IL-6 treatment were subjected to co-IP with anti-STAT3, followed by IB with anti-Trx-1 or anti-Kpnα1. (**F**) Trx-1 and STAT3 co-immunoprecipitate with Kpnα1. Lysates of HT-29 cells expressing Trx-1 without or with IL-6 treatment were subjected to co-IP with anti-Kpnα1 antibody, followed by IB with anti-STAT3 or anti-Trx-1. (**G**) Effect of Kpnα1 knockdown on protein interactions. Nuclear and cytosolic extracts of HT-29 cells transfected with Kpnα1 siRNA (siKpnα1) or negative control (NC) with 2 h IL-6 treatment were subjected to IB with anti-pSTAT3, anti-STAT3, anti-Trx-1, anti-Lamin B1 or anti-β-actin antibody.

To investigate the mechanism underlying IL-6-induced Trx-1 nuclear translocation, we performed a co-IP analysis in CRC cells to test whether endogenous Trx-1 interacts with pSTAT3. Lysates of HT-29 and SW480 cells without or with IL-6 treatment were immunoprecipitated with anti-Trx-1 antibody or anti-STAT3 and subjected to Western blot analysis. Interactions between pSTAT3 and Trx-1 protein were detectable and were enhanced by treatment with IL-6 (**Figure 3A-B**).

It has been reported that karyopherin α1 is directly linked to Trx-1 33 and also acts as an important mediator in regulating STAT3 nuclear import 34. The co-IP experiments also showed that endogenous karyopherin α1 is able to interact with Trx-1 and STAT3 simultaneously (**Figure 3A-B**), suggesting that karyopherin α1 mediates the interaction and complex formation of STAT3 and Trx-1. Subsequently, HT-29 cells expressing WT-Trx-1, MT-Trx-1, shTrx-1, or a control vector were also treated with IL-6 for 2 h, the cell lysates were immunoprecipitated with an anti-Trx-1 antibody, and the bound STAT3 and karyopherin α1 proteins were detected by Western blotting. As shown in **Figure C**, relative to the control group, WT-Trx-1 expression enhanced the interaction of Trx-1 with pSTAT3 and karyopherin α1, whereas MT-Trx-1 expression or shTrx-1 decreased that interaction even without IL-6 stimulation.

To further investigate whether the interaction of Trx-1 and STAT3 facilitates nuclear import, cytoplasmic and nuclear fractions of HT-29 cells untreated or treated with IL-6 were immunoprecipitated with anti-Trx-1 or anti-STAT3 antibodies. As shown in **Figure 3D and E**, treatment with IL-6 enhanced the interaction between STAT3 and Trx-1 in the nucleus but not in the cytoplasm. Moreover, endogenous karyopherin α1 was able to interact with Trx-1 and STAT3 simultaneously and those interactions were enhanced by IL-6 treatment (**Figure 3F**). The above results suggest that karyopherin α1 may mediate the IL-6-induced import of Trx-1 and STAT3 into the nucleus. Knockdown of karyopherin α1 did not affect the levels of IL-6, STAT3, and Trx-1 (**Figure S6**). However, silencing of karyopherin α1 markedly reduced the nuclear abundance of Trx-1 and pSTAT3 following IL-6 stimulation, indicating that this silencing prevented IL-6-induced Trx-1 and pSTAT3 nuclear import (**Figure 3G**). These results suggest an interaction between Trx-1, STAT3 and karyopherin α1, and that the complex so formed is required for IL-6 induced translocation of Trx-1 and pSTAT3 into the nucleus.

### Correlation of Trx-1 and STAT3 mRNA and nuclear protein expression in human CRC tissue

**Figure 4:**
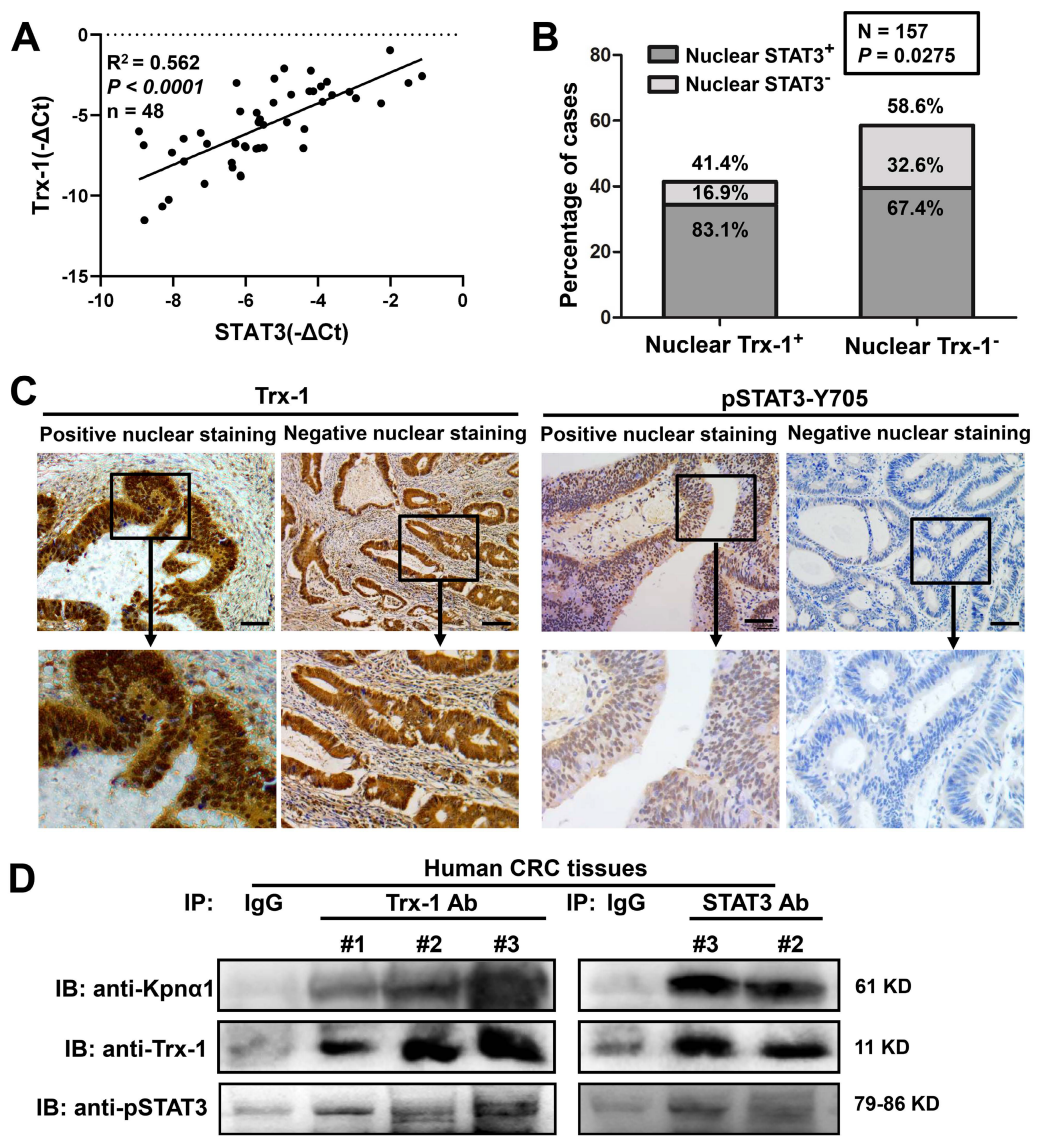
** Nuclear Trx-1 expression is increased in human colorectal cancer (CRC) tissue and is related to nuclear pSTAT3 expression.** (**A**) Correlative analysis of Trx-1 and STAT3 mRNA expressions in human CRC tissues (n = 48); mRNA levels were determined by qRT-PCR. The Spearman correlation coefficient with corresponding significance is indicated. (**B**) Correlation of nuclear Trx-1 and pSTAT3 expression by Chi-square analysis (*P* = 0.0275). (**C**) Nuclear immunohistochemistry of Trx-1 and pSTAT3 in human CRC tissue sections. Representative images of positive and negative staining in the nucleus are shown. Scale bar, 100 μm. (**D**) Co-IP of Trx-1, STAT3, and Kpnα1 in human CRC tissues. Tissue lysates were subjected to co-IP with anti-Trx-1 or anti-STAT3 antibody, followed by IB with anti-pSTAT3, anti-Kpnα1 or anti-Trx-1 antibody.

To determine whether Trx-1 levels in human CRC tissue are related to STAT3 expression, qPCR analysis was performed on tumor tissues from 48 CRC patients. As shown in **Figure 4A**, Trx-1 mRNA expression was associated with STAT3 mRNA expression (*R*^2^ = 0.562, *P* < 0.01). We then performed immunohistochemical analysis of consecutive sections of human CRC tissue to assess the nuclear expression of Trx-1 and pSTAT3, which showed a significant positive correlation (*P* = 0.0275, **Table S4 and Figure 4B-C**). Co-IP analysis also confirmed the interaction of endogenous Trx-1, pSTAT3, and karyopherin α1 in human CRC tissue (**Figure 4D**). We further investigated the relationship of nuclear expression of Trx-1 with patients' pathological and clinical parameters (**Table S5**). A higher proportion of positive Trx-1 nuclear staining was observed in the distal colon and rectum (*P* < 0.05); additionally, patients with lymph node metastases (*P* < 0.01) and distant metastases (*P* < 0.05) had a higher percentage of nuclear Trx-1 staining than those without metastases. These results suggest that nuclear Trx-1 expression may play an important role in the progression of CRC.

### Treatment with PX-12 attenuates tumor progression in the AOM/DSS-induced mouse model of CAC

PX-12 is a competitive, irreversible inhibitor of Trx1 35. To determine whether treatment with PX-12 prevents IL-6-induced Trx-1 nuclear translocation in CRC cells, HT-29 cells were pre-treated with 5 μM PX-12 for 24 h and then treated with IL-6 (20 ng/mL) for 2 h. Expression of Trx-1 and pSTAT3 in cytoplasmic and nuclear fractions was analyzed by Western blotting. As shown in **Figure S7,** treatment with PX-12 decreased Trx-1 and pSTAT3 levels in the nucleus of HT-29 cells after IL-6 stimulation.

**Figure 5:**
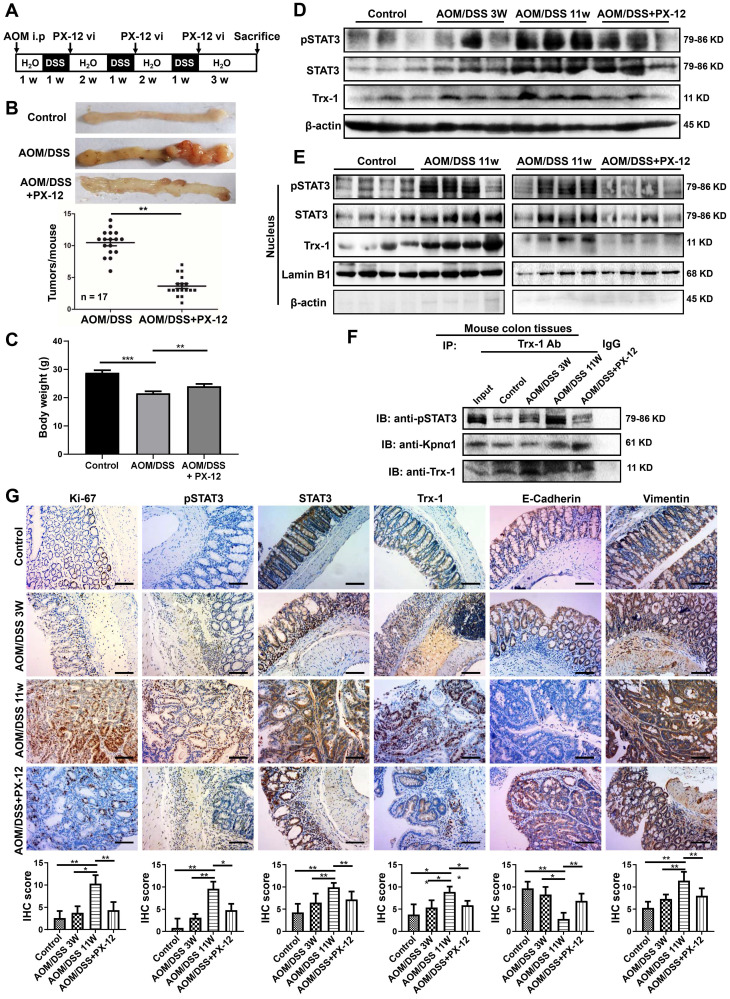
** Trx-1 inhibitor PX-12 inhibits the progression of colitis-associated tumorigenesis in a mouse model of colitis-associated colorectal cancer (CAC).** (**A**) Schematic overview of the AOM/DSS mouse model and administration of PX-12 as described in Methods. (**B**) Treatment with PX-12 reduced the number of colitis-associated tumors in AOM/DSS model mice. Mice were exposed to AOM/DSS or AOM/DSS plus PX-12 treatment for 11 weeks. Images show the representative macroscopic morphology of the colon with tumors in the negative control, AOM/DSS model, and PX-12 treatment groups. Counts represent the number of tumors formed in each mouse colon (n = 17 per group). (**C**) Body weight of mice exposed to AOM/DSS treatment or AOM/DSS plus PX-12 treatment for 11 weeks. (**D**) Western blot analysis of the indicated proteins in the colon tissues of the control group, AOM/DSS model mice after 3 weeks, AOM/DSS model mice after 11 weeks, and AOM/DSS model mice with PX-12 treatment. (**E**) Western blotting of nuclear extracts of colon tissues from the control group, AOM/DSS model mice, and AOM/DSS model mice with PX-12 treatment (n = 4 from each group). Blots were probed with anti-pSTAT3, anti-STAT3, anti-Trx-1, or Lamin B1. (**F**) Immunoprecipitation (IP) of interacting proteins from lysates of mouse colon tissues from the control group, AOM/DSS model mice, and AOM/DSS model mice with PX-12 treatment. IP was performed with anti-Trx-1 antibody or control IgG, and followed by IB with anti-pSTAT3, anti-Kpnα1 or anti-Trx-1 antibody. (**G**) Protein expression in the distal colon sections of mice, determined by immunohistochemistry for the indicated proteins. Scale bars, 100 μm. *P < 0.05, **P < 0.01, ***P < 0.001.

We then applied PX-12 to investigate the role of nuclear Trx-1 expression in the development of CRC, using the well-established AOM/DSS model of murine CRC. The schedule for the AOM/DSS mouse model and PX-12 treatment is shown in **Figure 5A**. After AOM/DSS treatment for 11 weeks, the mice showed obvious colitis and spontaneous tumor formation in the colon with loss of body weight (**Figure 5B-C and Figure S8**). Administration of PX-12 during CAC induction significantly reduced the incidence of tumor formation (**Figure 5B**) as well as body weight loss (**Figure 5C**). In addition, Western blot analysis of colon tissue showed that the protein expression of pSTAT3, STAT3, and Trx-1 was increased in the AOM/DSS mice, whereas administration of PX-12 resulted in expression decreases (**Figure 5D**). We performed Western blot analysis of Trx-1 and STAT3 expressions using cytosolic and nuclear fractions from colon tissue. Treatment with AOM/DSS increased the nuclear expression of Trx-1 and pSTAT3, which increases were prevented by treatment with PX-12 (**Figure 5E**). Co-IP showed that Trx-1 bound pSTAT3 and karyopherin α1 (**Figure 5F**), and the interaction of Trx-1 with pSTAT3 was increased with AOM/DSS treatment but decreased with PX-12 treatment. Histological analysis of the distal colon and rectum in CAC mice further showed PX-12 administration to reduce tumor cell proliferation (fewer Ki-67-positive cells) (**Figure 5G**). In addition, IHC analysis showed PX-12 administration to reverse AOM/DSS induction of STAT3, pSTAT3, and Trx-1 expression and EMT (**Figure 5G**). These results suggest that nuclear Trx-1 expression is involved in colitis-associated cancer progression, and that the Trx-1 inhibitor PX-12 suppresses EMT and tumorigenesis progression in colitis-associated tumors.

### Inhibition of AOM/DSS-induced CAC progression in *Txn1*(KK81-82EE) knockin mice

**Figure 6:**
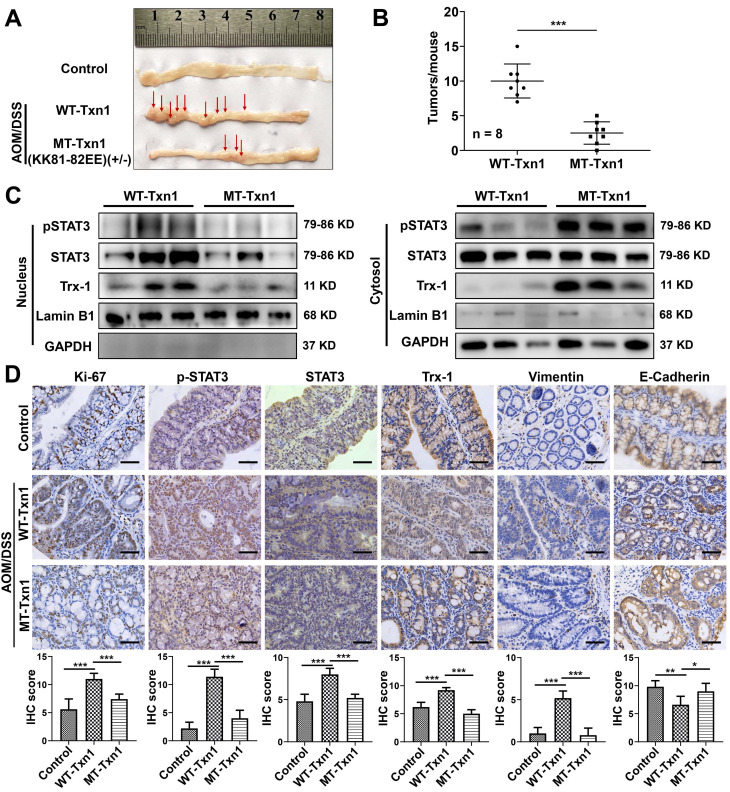
** Nuclear translocation of Trx-1 plays an important role in colitis-associated colon cancer.** (**A**) Representative images of macroscopic colon morphology in wild-type mice and mutant *Txn1*(KK81-82EE)-knockin mice treated with AOM/DSS to induce colitis-associated cancer. (**B**) The number of tumors formed in each mouse colon. (**C**) Cytoplasmic and nuclear levels of pSTAT3, STAT3, and Trx-1 in colon tissue, determined by Western blotting. (**D**) Protein expression in the distal colon sections of mice, determined by immunohistochemistry for the indicated proteins. Scale bars, 50 μm. *P < 0.05, **P < 0.01, ***P < 0.001.

Knockin mice with the *Txn1*(KK81-82EE) mutation were generated via CRISPR/Cas9 (**Figure S9**). As few homozygous-knockin mice were produced, heterozygous mice were used in experiments. To clarify the role of Trx-1 nuclear translocation in colitis-associated colorectal cancer, *Txn1*(KK81-82EE)(+/-) and wild-type controls were treated with AOM/DSS and tumor formation was examined (**Figure 6A**). Wild-type mice exhibited a higher tumor burden than *Txn1*(KK81-82EE)(+/-) mice (**Figure 6A-B**). Western blotting analysis of colon tissues showed that Trx-1 mainly localized to the nucleus in treated wild-type mice, whereas it mainly localized to the cytoplasm in *Txn1*(KK81-82EE)(+/-) mice (**Figure 6C**). Nuclear pSTAT3 expression was also decreased in *Txn1*(KK81-82EE)(+/-) mice compared with the wild-type (**Figure 6C**). IHC analysis showed that *Txn1*(KK81-82EE) reversed AOM/DSS-induced increases in Ki-67, pSTAT3, and Trx-1 expression, as well as EMT (**Figure 6D**). These data support the idea that translocation of Trx-1 to the nucleus plays an important role in promoting tumorigenesis in the AOM/DSS model.

## Discussion

Metastasis is the leading cause of CRC-related mortality. Elucidating the molecular mechanisms and finding an effective treatment for CRC metastasis will help improve the prognosis of patients with metastatic CRC. We have previously shown that Trx-1 expression is upregulated in CRC tissues and significantly correlates with clinical stage, lymph node metastasis and poor survival 26. In this study, we identified novel and important functions of Trx-1 nuclear translocation in various aspects of CRC progression; specifically, we demonstrated that nuclear translocation of Trx-1 promotes EMT and metastasis of CRC by enhancing IL-6/STAT3 signaling through interaction with STAT3* in vitro* and *in vivo*.

STAT3 is a dominant signaling pathway downstream of IL-6 signaling 14. Sustained activation of STAT3 in the nucleus is considered a hallmark of many human tumor types and leads to upregulation of a number of growth-promoting genes that contribute to tumor aggressiveness 16, 36. Recent studies have confirmed that intracellular Trx-1 is translocated to the nucleus in response to environmental stimuli, including oxidative stress 20, 37, 38. Trx-1 is considered an anti-oxidant, anti-apoptotic effector, and redox regulator in signal transduction in response to H_2_O_2_ stimulation 20, 39. Our results showed Trx-1 nuclear translocation to be induced by treatment with IL-6, and that translocation to be suppressed after knockdown of STAT3 or specific deactivation of STAT3 phosphorylation. Moreover, overexpression of Trx-1 resulted in increased nuclear translocation and expression of pSTAT3 following IL-6 treatment, while knockdown of Trx-1 or inhibition of Trx-1 nuclear translocation via mutation (*Trx-1* KK81-82EE) abolished IL-6-induced pSTAT3 nuclear import. Finally, inhibition of Trx-1 nuclear translocation prevented the IL-6-induced migration, invasion, and EMT of CRC cells. These results suggest that nuclear translocation of Trx-1 functions as an effector in the IL-6/STAT3-triggered signaling pathway and also regulates endogenous IL-6/STAT3 signaling.

We also found Trx-1 to interact with STAT3 and translocated into the nucleus in CRC cells treated with IL-6. Karyopherin α1 has been reported to bind specific pSTAT3 domains and translocate to the nucleus after stimulation by cytokines 40. It has also been reported to directly associates with Trx-1 when H_2_O_2_ triggers Trx-1 nuclear import 32. Therefore, the question arose as to whether karyopherin α1 is associated with the interaction between Trx-1 and STAT3 and their nuclear translocation. Our results confirmed an interaction between Trx-1, STAT3 and karyopherin α1; in addition, silencing of karyopherin α1 prevented this interaction and the nuclear translocation of Trx-1 and STAT3, suggesting that karyopherin α1 specifically mediates IL-6 induced translocation of the Trx-1-pSTAT3 complex to the nucleus.

In normal cells, Trx-1 is mainly located in the cytoplasm; however, it was detected in nuclei among 76% of samples from the invasion front of gallbladder carcinomas and 63.3% of breast cancer tissues 41, 42. There is an association between positive Trx-1 nuclear staining and lower histological grading and poorer prognosis in both of these cancers 41, 42. In the present study, we used IHC to analyze the nuclear localization of Trx-1 and pSTAT3 in 157 human CRC tissues. Nearly 42% of human CRC tissues had moderate to strong staining of Trx-1 in the nucleus, and nuclear expression of Trx-1 appeared to be closely correlated with lymph node metastasis and distant metastasis. We also investigated the correlation between Trx-1 and pSTAT3 levels in human CRC tissues by qPCR and IHC, which showed Trx-1 mRNA expression to be associated with that of STAT3 and nuclear staining of Trx-1 to have significant positive correlation with nuclear staining of pSTAT3. Co-IP analysis also confirmed the interaction of endogenous Trx-1, pSTAT3, and karyopherin α1 in human CRC tissue.

Furthermore, treatment with the Trx-1 inhibitor PX-12 significantly impairs STAT3 activation and suppresses the development of AOM/DSS-induced CAC in mice. PX-12 is currently in clinical development as an anti-tumor agent via specific inhibition of Trx-1 43, 44. We have previously observed that PX-12 inhibits the growth of CRC cells, induces apoptosis, and reduces cell migration and invasion 45. The IL-6/STAT3 signaling pathway is thought to be linked to inflammation-associated tumorigenesis, as occurs in CAC 10, 13, and the AOM/DSS induced mouse model is generally accepted as an ideal model for studying the development of colon tumorigenesis 46. In this study, we used this model and found that nuclear Trx-1 and pSTAT3 protein levels were increased in AOM/DSS-induced CAC mouse, but decreased with PX-12 treatment, suggesting that PX-12 can suppress the progression of CAC by blocking nuclear translocation of Trx-1 and pSTAT3. *In vitro* experiments in HT-29 cells also showed PX-12 to suppress IL-6-induced translocation of Trx-1 and pSTAT3.

To elucidate the role of Trx-1 nuclear translocation in colitis-associated cancer *in vivo*, we also generated *Txn1*(KK81-82EE)-knockin mice using CRISPR/Cas9 technology. We found that the AOM/DSS-induced nuclear localization of Trx-1 was suppressed in *Txn1*(KK81-82EE) mice. The* Txn1*(KK81-82EE) mutation also decreased the expression of pSTAT3 and Ki-67, and inhibited EMT and cancer progression in AOM/DSS-induced CAC. These data support the premise that translocation of Trx-1 to the nucleus is important in promoting tumorigenesis in AOM/DSS-induced CAC.

Overall, our results show that nuclear translocation of Trx-1 plays a key role in the development of CRC by activating the IL-6-STAT3 signaling pathway. In particular, we have shown that this translocation is required for IL-6-induced EMT and metastasis of CRC. Furthermore, we determined that Trx-1 and STAT3 form a functional complex with karyopherin α1, translocate into the nucleus, and then promote the progression of CRC. Therefore, the interaction between Trx-1 and STAT3 represents a novel, critical mechanism for the control of IL-6 signaling and CRC development and metastasis. Our results provide important insights into the mechanisms of IL-6-triggered STAT3 activation and identify nuclear translocation of Trx-1 as a potential therapeutic target for the treatment of CRC and CAC.

## Supplementary Material

Supplementary methods, figures and tables.

## Abbreviations

AOM: azoxymethane
CAC: colitis-associated cancer
Co-IP: Co-immunoprecipitation
CRC: colorectal cancer
DSS: dextran sodium sulfate
EMT: epithelial-mesenchymal transition
H_2_O_2_: hydrogen peroxide
IBD: inflammatory bowel disease
IL-6: interleukin-6
Kpnα1: karyopherin α1
MT-Trx-1: KK81-82EE mutant Trx-1 gene
pSTAT3^Y705^: STAT3 phosphorylation of the residue Y705
PX-12: 1-methylpropl 2-imidazolyl disulfide
Trx-1: thioredoxin-1
UC: ulcerative colitis
WT-Trx-1: wild-type Trx-1 gene
