# Supplementary material for: Nuclear translocation of thioredoxin-1 promotes colorectal cancer development via modulation of the IL-6/STAT3 signaling axis through interaction with STAT3
Source: Theranostics. 2023 Aug 28;13(14):4730–44. doi: 10.7150/thno.85460 (PMC10526669; doi:10.7150/thno.85460)
Supplement: Supplementary file 1 — Supplementary methods, figures and tables. [file thnov13p4730s1.pdf]

Supplementary Materials for

Nuclear translocation of thioredoxin-1 promotes colorectal cancer development via modulation of the IL-6/STAT3 signaling axis through interaction with STAT3

This PDF file includes:

- Figures S1 to S9
- Tables S1 to S5
- Supplementary methods

Supplementary data

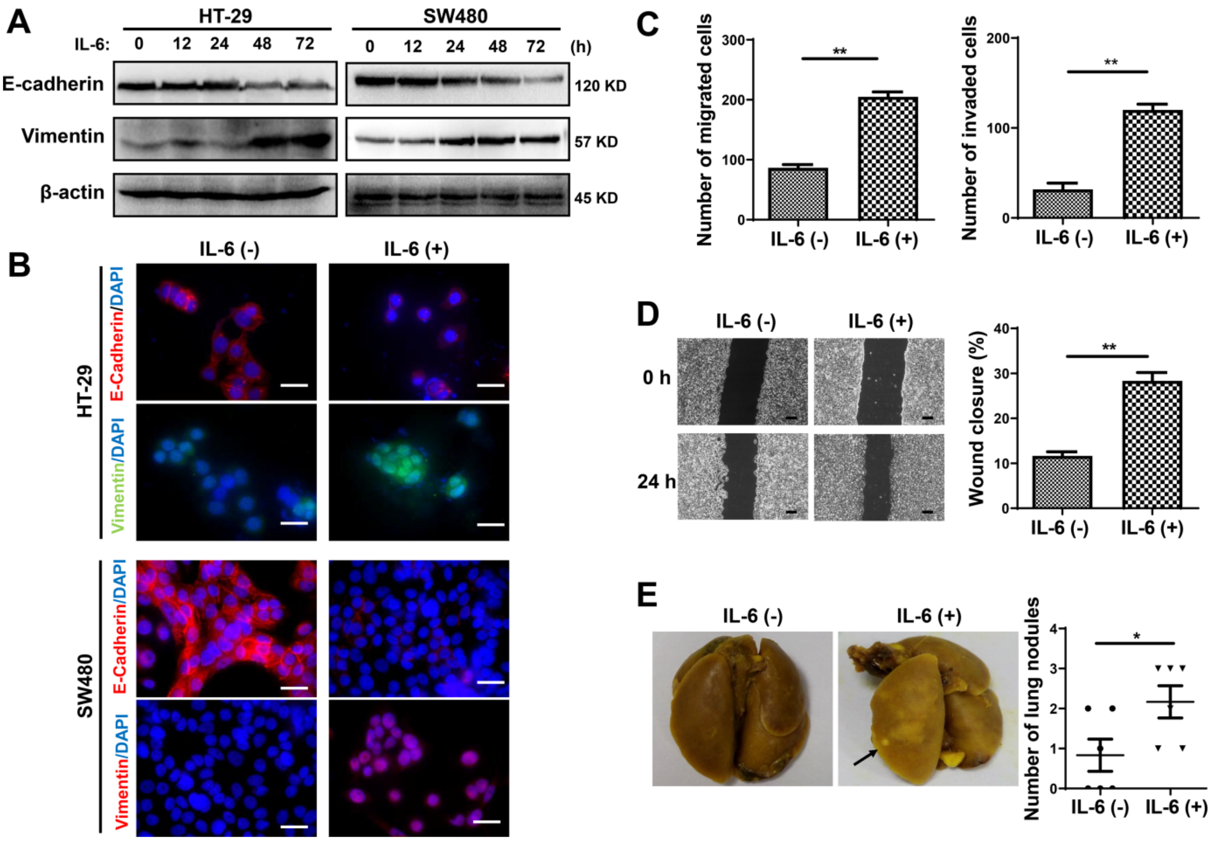

Supplementary Figure S1 IL-6 induces migration, invasion, and epithelial-to-mesenchymal transition (EMT) of colorectal cancer cells in *vitro* and promotes metastasis of colorectal cancer cells *in vivo*. (A) Western blot analysis of E-cadherin and vimentin proteins after treatment of 20 ng/mL IL-6 for indicated periods. (B) Immunofluorescence staining of E-cadherin and vimentin in HT-29 and SW480 cells treated with 20 ng/mL IL-6 for 72 h (nuclei stained with DAPI). Scale bar, 20 μm. (C) Migration and invasion of SW480 cells treated with IL-6 (20 ng/mL) for 48 h were detected by Transwell assay. The number of migrated and invaded cells is shown in the histogram. (D) Wound healing (scratch) assay of HT-29 cells treated with IL-6 (20 ng/mL) for 24 h. (E) Formation of lung metastases 8 weeks after tail-vein injection of control and IL-6-pretreated (5 days) SW480 cells in NOD/SCID mice. Representative images of the entire lungs and quantification of lung microscopic

nodules per mouse are shown (n = 6 from each group). Error bars represent S.E.M. *$P < 0.05$, *$P < 0.01$.

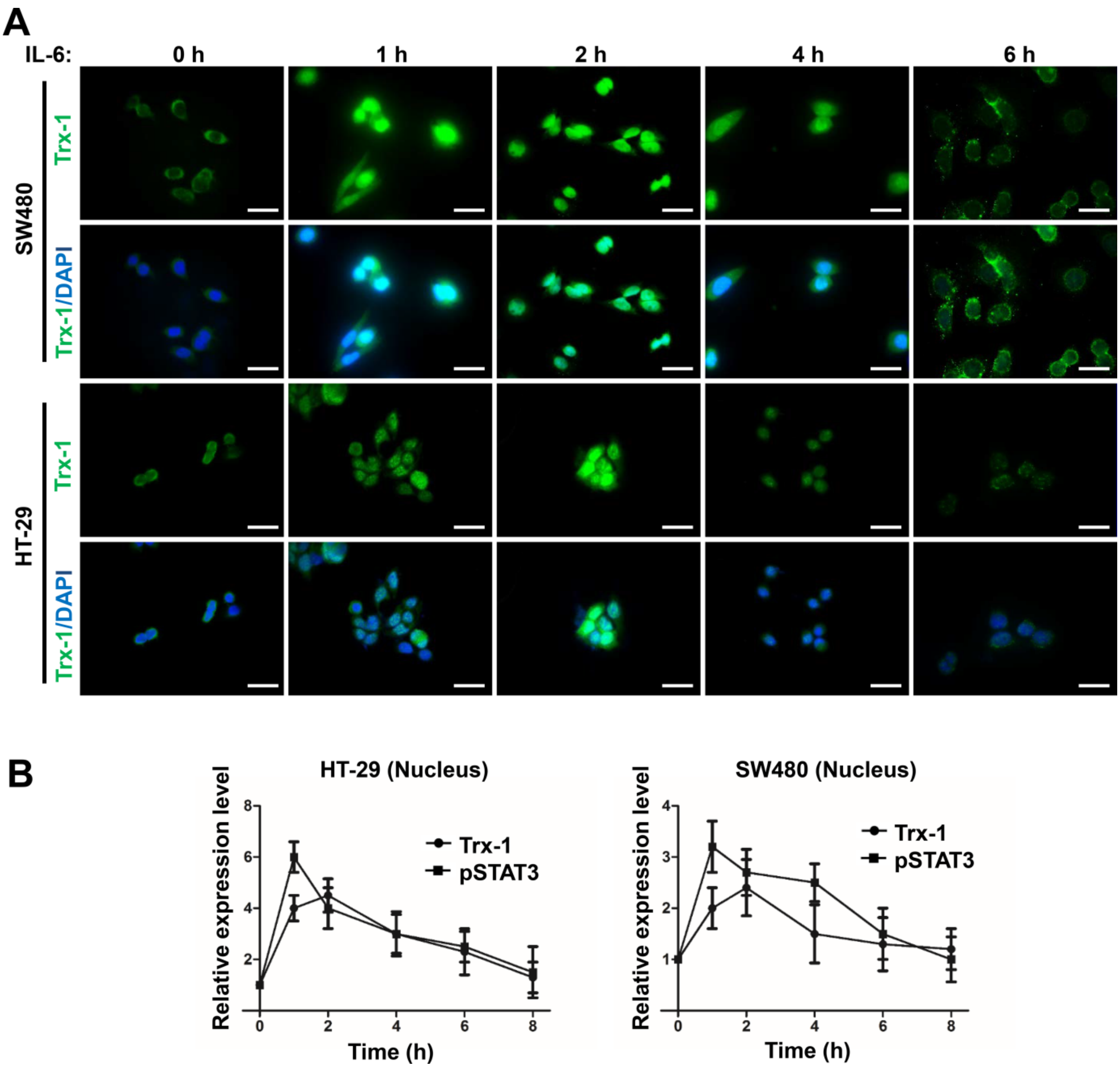

Supplementary Figure S2 Treatment with IL-6 results in changes in nuclear Trx-1 and pSTAT3 protein levels. (A) Immunofluorescence staining of Trx-1 in HT-29 and SW480 cells treated with 20 ng/mL IL-6 for the indicated periods (nuclei stained with DAPI). Scale bar, 20 μm. (B) The relative levels of nuclear Trx-1 and pSTAT3 in HT-29 and SW480 cells treated with 20 ng/mL IL-6 for the indicated time periods.

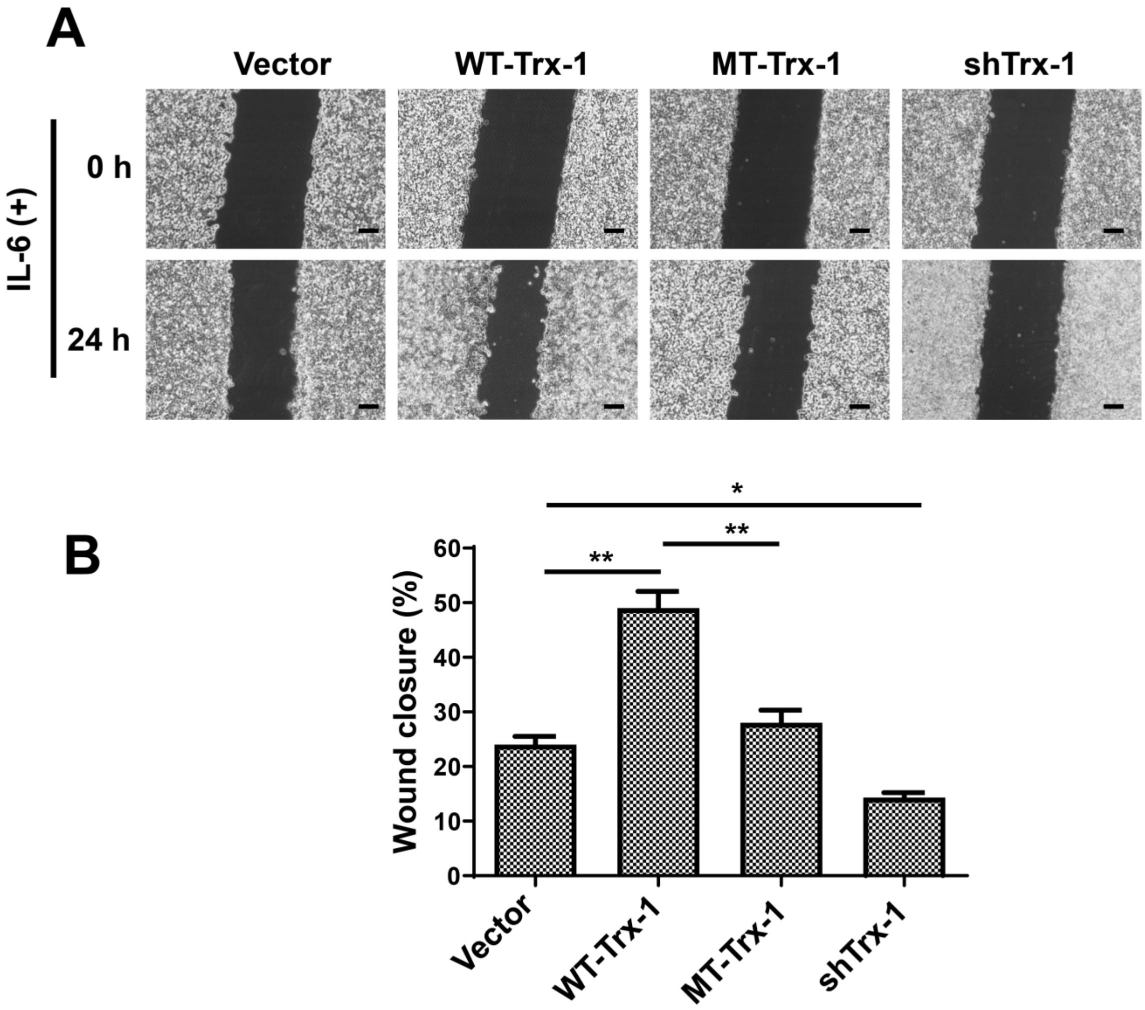

Supplementary Figure S3 Nuclear translocation of Trx-1 is required for IL-6-induced CRC cell migration. (A) Wound healing (scratch) assay of HT-29 cells expressing control vector, WT-Trx-1, MT-Trx-1, or shTrx-1 treated with IL-6 (20 ng/mL) for 24 h. (B) Percentage of relative wound closure after 24 h. Scale bar, 100 μm. ** $P < 0.01$, *$P < 0.05$.

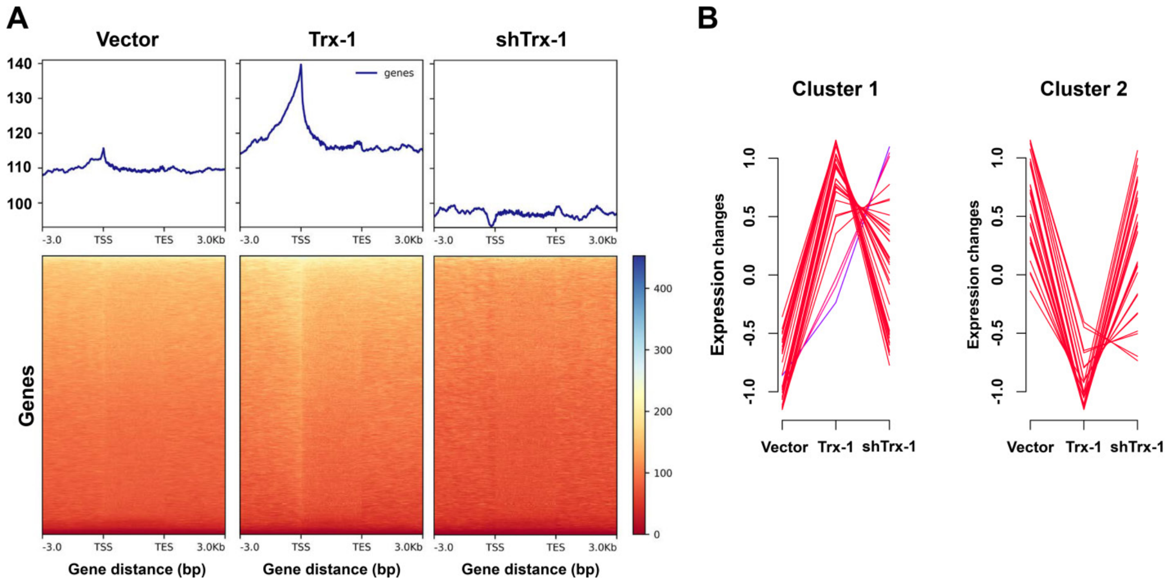

Supplementary Figure S4 Genome-wide Trx-1-induced changes in STAT3 occupancy. STAT3 ChIP-seq and RNA-seq were performed in SW480 cells transfected with control-vector, Trx-1, or shTrx-1, and treated with IL-6 (20 ng/mL) for 2 h. (A) Signal of ChIP-seq peaks 3 kb upstream and downstream of transcription start sites (TSS). (B) Clusters of genes with similar expression patterns. The associated genes with differential peaks among three comparisons in ChIP-seq were overlapped with the genes with differential expression in RNA-seq between cells transfected with Vector and Trx-1 to obtain the target genes. The expression patterns of the target genes were analysed using the Mfuzz R package. Based on the similarity of the expression patterns, two clusters were identified, showing that changes in the STAT3 ChIP-seq coverage often lead to transcription alteration.

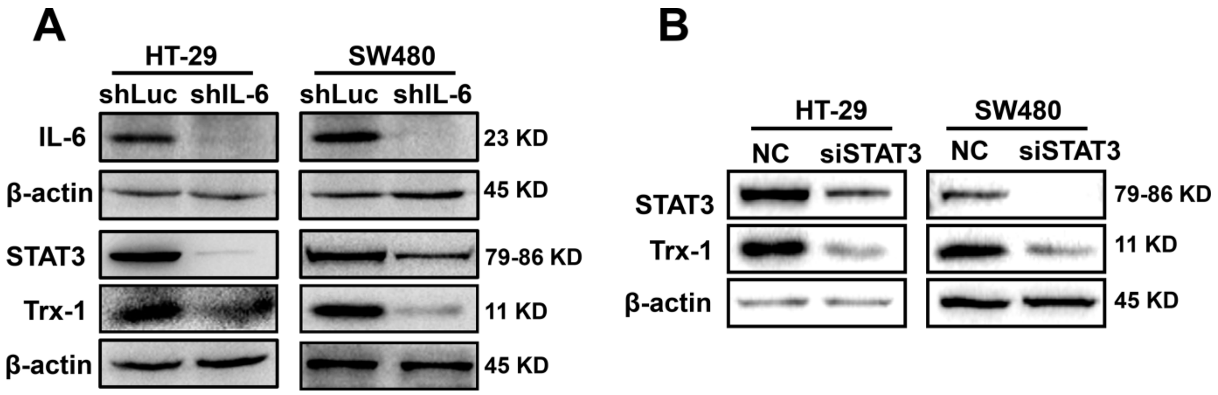

Supplementary Figure S5 Knockdown of IL-6 or STAT3 suppresses Trx-1 expression. (A) Western blot analysis of IL-6, STAT3 and Trx-1 expression in HT-29 and SW480 cells transduced with lenti-shIL-6 or lenti-shLuc (control). (B) Western blot analysis of STAT3 and Trx-1 expression in HT-29 and SW480 cells transfected with siSTAT3 or negative control (NC) for 48 h.

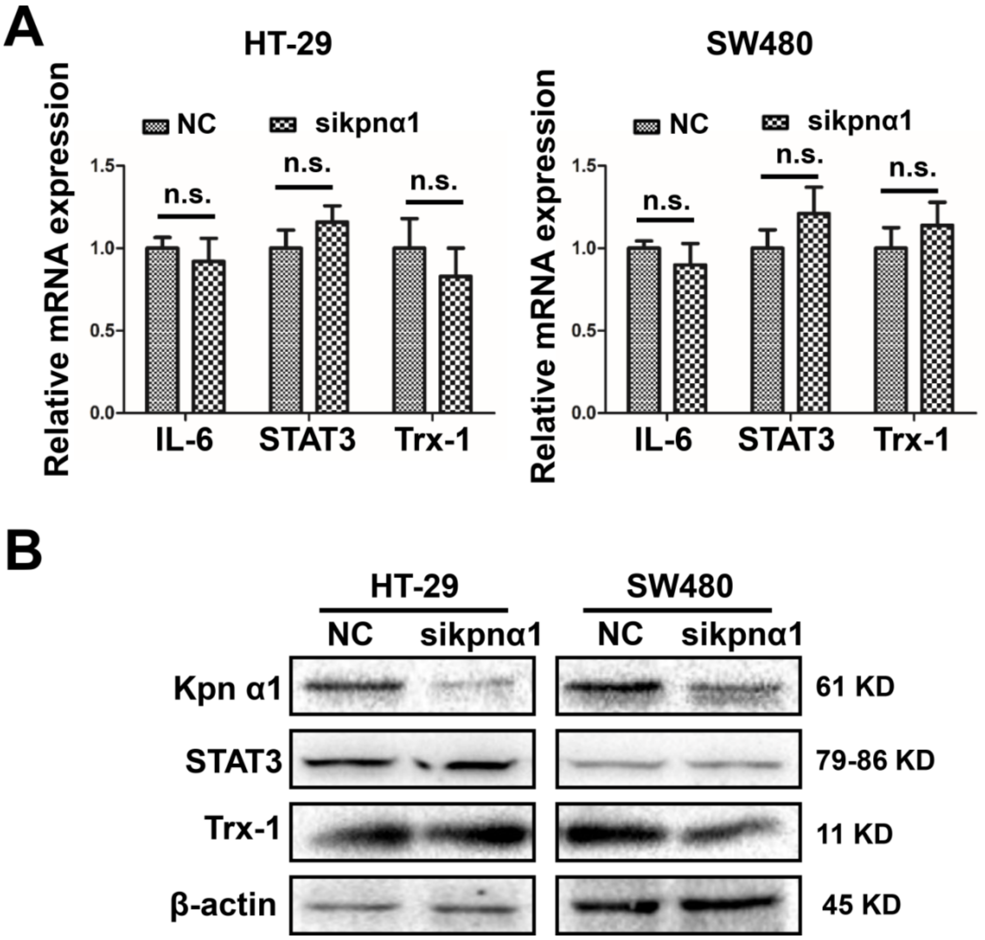

Supplementary Figure S6 The effect of Karyopherin α1 (Kpnα1) knockdown on STAT3 and Trx-1 expressions. (A) Real-time PCR analysis of IL-6, STAT3 and Trx-1 expression in HT-29 and SW480 cells transfected with Karyopherin α1 siRNA (siKpnα1) or negative control (NC) for 48 h. (B) Western blot analysis of STAT3 and Trx-1 expression in HT-29 and SW480 cells transfected with siKpnα1 or NC for 48 h. ns, not significant.

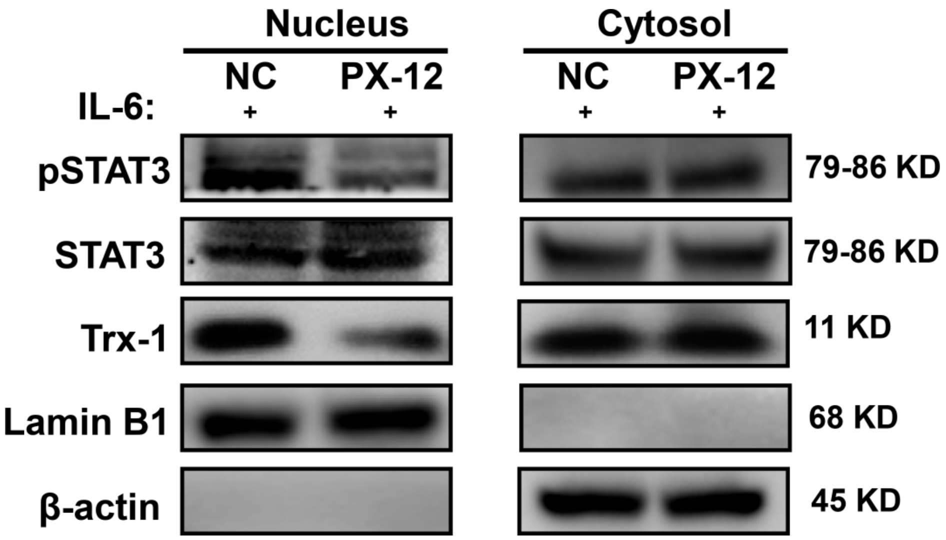

Supplementary Figure S7 PX-12 treatment induces a decrease of nuclear pSTAT3 and Trx-1 expressions in HT-29 cells treated with IL-6 for 2 h.

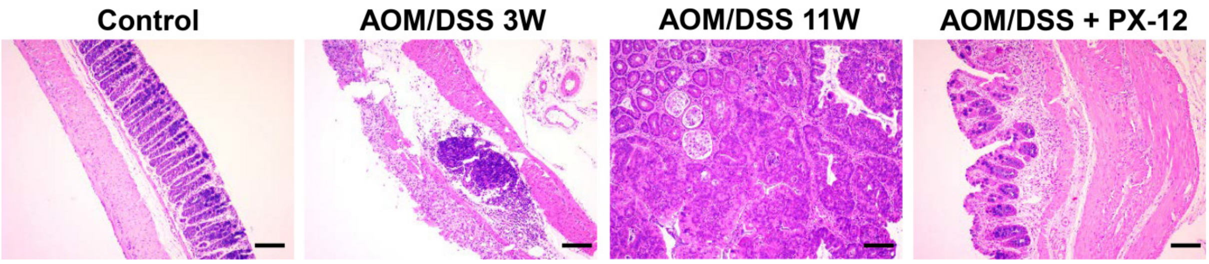

Supplementary Figure S8 H&E-stained sections of colons from control group, AOM/DSS model mice at 3 week, AOM/DSS model mice at 11 week and AOM/DSS model mice with PX-12 treatment. Scale bars, 100 μm.

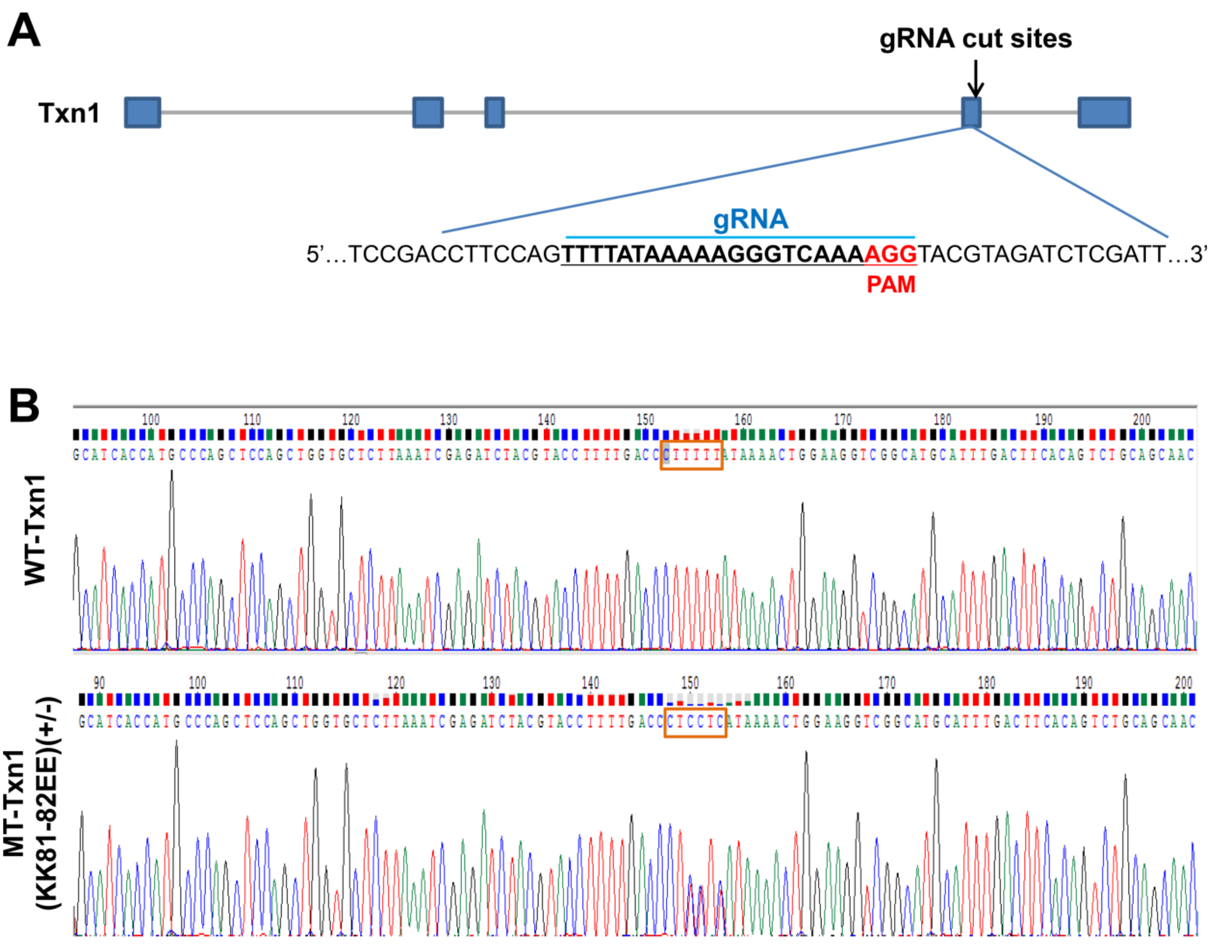

Supplementary Figure S9 Generation of mutant Txn1(KK81-82EE)-knockin mice via CRISPR/Cas9 technology. (A) Diagram of mouse Txn1 exon4 gRNA. The gRNA sequence is underlined. The sequence of the protospacer adjacent motif (PAM) is indicated in red. (B) Txn1 genomic sequence of F1 generation mutant (MT) Txn1 (KK81-82EE)-knockin and wild-type (WT) mice. (Top) DNA sequences of WT mouse. (Bottom) DNA sequences of MT-Txn1 (KK81-82EE)-knockin (+/-), three point mutations (AAAAAG to GAGGAG) in one allele.

Supplementary Table S1 Primers used for real-time PCR analysis

Gene	Primer sequence (5’-3’)
GAPDH forward:	CCAGCCGAGCCACATCGCTC
GAPDH reverse:	ATGAGCCCCAGCCTTCTCCAT
IL-6 forward:	GGTACATCCTCGACGGCATCT
IL-6 reverse:	GT GCCTCTTTGCTGCTTTCAC
STAT3 forward:	GGACTTCCCGGACAGTGAG
STAT3 reverse:	ATCGCTTGTGTTGCCAGAG
Trx-1 forward:	CAACCCTTTCTTTCATTCCCTCT
Trx-1 reverse:	CACCCACCTTTTGTCCCTTCT

Supplementary Table S2 The nuclear/cytosolic Trx-1 ratio after IL-6 treatment for the indicated time

IL-6 (20 ng/mL) stimulation	nuclear/cytosolic Trx-1 ratio	
	HT-29 cells	SW480 cells
0 h	1	1
1 h	8	5
2 h	10	6.8
4 h	2.13	1.83
6 h	0.95	1.1
8 h	0.5	1.5

Supplementary Table S3 Lung metastasis nodules in a xenograft mouse model with SW480 cells stably expressing WT-Trx-1 or MT-Trx-1

	Days	Mice with metastasis	Number of lung metastasis nodules		
			< 0.5 mm	> 0.5 mm	Total
WT-Trx-1	56	5/6	28	10	38
WT-Trx-1+IL-6	56	6/6	44	32	76
MT-Trx-1	56	1/6	9	1	10
MT-Trx-1+IL-6	56	2/6	16	3	19

Supplementary Table S4 Correlation of Trx-1 and pSTAT3 nuclear expression in 157 human colorectal cancer tissues

			pSTAT3 nuclear staining		*p* value
		Cases	Negative	Positive	
	Cases		41	116	
Trx-1 nuclear staining	Negative	92	30 (73%)	62 (53%)	**0.0275***
	Positive	65	11 (27%)	54 (47%)	

*$P < 0.05$.

Supplementary Table S5 Association of Trx-1 nuclear expression and clinic pathological parameters in human colorectal cancer tissues

Variables	Trx-1 nuclear staining			*p* values
	All cases (n = 157)	Negative (n = 92)	Positive (n = 65)	
Age(year)				
< 65	58	34	24	0.997
≥ 65	99	58	41	
Clinical stage				
I-II	69	40	29	0.88
III-IV	88	52	36	
pN Status				
N0	75	58	17	< **0.001*** **
N1-N2	82	34	48	
Metastasis				
M0	126	81	45	**0.004***
M1	31	11	20	
Tumor site				
Proximal	30	19	11	**0.034***
Colon distal	54	38	16	
Colon rectum	73	35	38	

*$P < 0.05$, **$P < 0.01$, ***$P < 0.001$.

Supplementary methods

Wound healing scratch assay

The cells studied were cultured for 24 h, and a scratch was introduced into the confluent cell layer with a 200 μl pipette tip. Cells were washed three times with medium to remove detached cells and then incubated with IL-6 for 24 h. Photographs were taken at each time point under a phase contrast microscope (Olympus).

Metastasis in a xenograft mouse model

Male NOD/SCID mice (6-8 weeks old) were provided by Shanghai Slaccas Animal Center. SW480 cells expressing the wild type Trx-1 gene (SW480-WT-Trx-1) or mutant Trx-1 (SW480-MT-Trx-1) were pretreated with vehicle or IL-6 (20 ng/mL) for 5 days, and then the cells (4×10^6/0.2 mL) were injected into the tail vein of NOD/SCID mice. Ten weeks after injection into the tail vein, the mice were sacrificed, and the metastatic nodules in the lungs were examined. All experiments were approved by the Animal Experimental Ethics Committee of Wenzhou Medical University.
